# Lumbosacral fixation using sacroiliac buttress screws: a modification to the Jackson technique with intrasacral rods

**DOI:** 10.1186/1748-7161-9-8

**Published:** 2014-07-12

**Authors:** Kentaro Fukuda, Masakazu Takemitsu, Masafumi Machida, Takashi Asazuma

**Affiliations:** 1 Department of Orthopedic Surgery, National Hospital Organization, Murayama Medical Center, 2-37-1, Gakuen, Musashimurayama, Tokyo 208-0011, Japan; 2 Department of Orthopedic Surgery, Saiseikai Yokohamashi Tobu Hospital, 3-6-1, Shimosueyoshi, Tsurumi-ku, Yokohama 230-8765, Japan

**Keywords:** Posterior lumbosacral fixation, Lateral sacral mass, Iliac buttress, Intrasacral screw, Surgical technique

## Abstract

**Background:**

The use of intrasacral rods has been previously reported for posterior lumbosacral fixation. However, problems associated with this technique include poor stability of the rod in the sacrum, difficulty in contouring the rod to fit the lateral sacral mass, and the complicated assembly procedure for the rod and pedicle screws in the thoracolumbar segments after insertion of the rod into the sacrum.

**Methods:**

We used a screw with a polyaxial head instead of an intrasacral rod, which was inserted into the lateral sacral mass and assembled to the rod connected cephalad to pedicle screws. The dorsal side of the screw was stabilized by the sacral subchondral bone at the sacroiliac joint with iliac buttress coverage, and the tip of the screw was anchored by the sacral cortex.

**Results:**

Three different cases were used to illustrate lumbosacral fixation using intrasacral screws as an anchor for the spinal instrumentation. Effective resistance of flexural bending moment and fusion were achieved in these patients at the lumbosacral level.

**Conclusions:**

An intrasacral screw can be stabilized by subchondral bone with iliac buttress coverage at the dorsal and ventral sacral cortex. Posterior spinal fusion with this screw technique enables easier assembly of the instrumentation and presents better stabilization than that provided by the previously reported intrasacral rod technique for correction and fusion of thoracolumbar kyphoscoliosis.

## Background

Posterior spinal fusion at the lumbosacral junction remains challenging because this surgery is associated with a high rate of complications, such as pseudarthrosis and instrumentation failure [1,2], particularly in patients requiring correction for long fusion segments and/or severe deformities. Bilateral bicortical screws at the S1 segment are not sufficient as distal foundations for long fusions; thus, an additional anchor at the sacrum and/or ilium is required. Several surgical techniques have been developed to overcome this problem, including the use of iliac rods or screws [3-5], iliosacral screws [6], and intrasacral rods [7].

Jackson and McManus [7] reported that the placement of intrasacral rods is a useful technique for posterior lumbosacral fixation, in which rods are inserted into the lateral sacral mass and attached to segmental pedicle screws. The rods are stabilized by the subchondral bone of the sacrum at the sacroiliac joint with iliac buttress coverage. This buttress effect provides resistance to flexural bending at the lumbosacral level. One of the difficulties of this technique is in contouring rod to fit the lateral sacral mass and spinal curvature. The procedure of attaching the rod to the S1 pedicle screw and every level cephalad is technically demanding, particularly in patients with severe spinal deformities. To overcome this difficulty, we modified the methods of Jackson and McManus [7] by way of using a screw with a polyaxial head instead of the intrasacral rod for a good distal foundation and easy assembly of the instrumentation. In this report, we introduce a surgical technique based on a modification to the Jackson procedure.

## Methods

**Figure 1:**
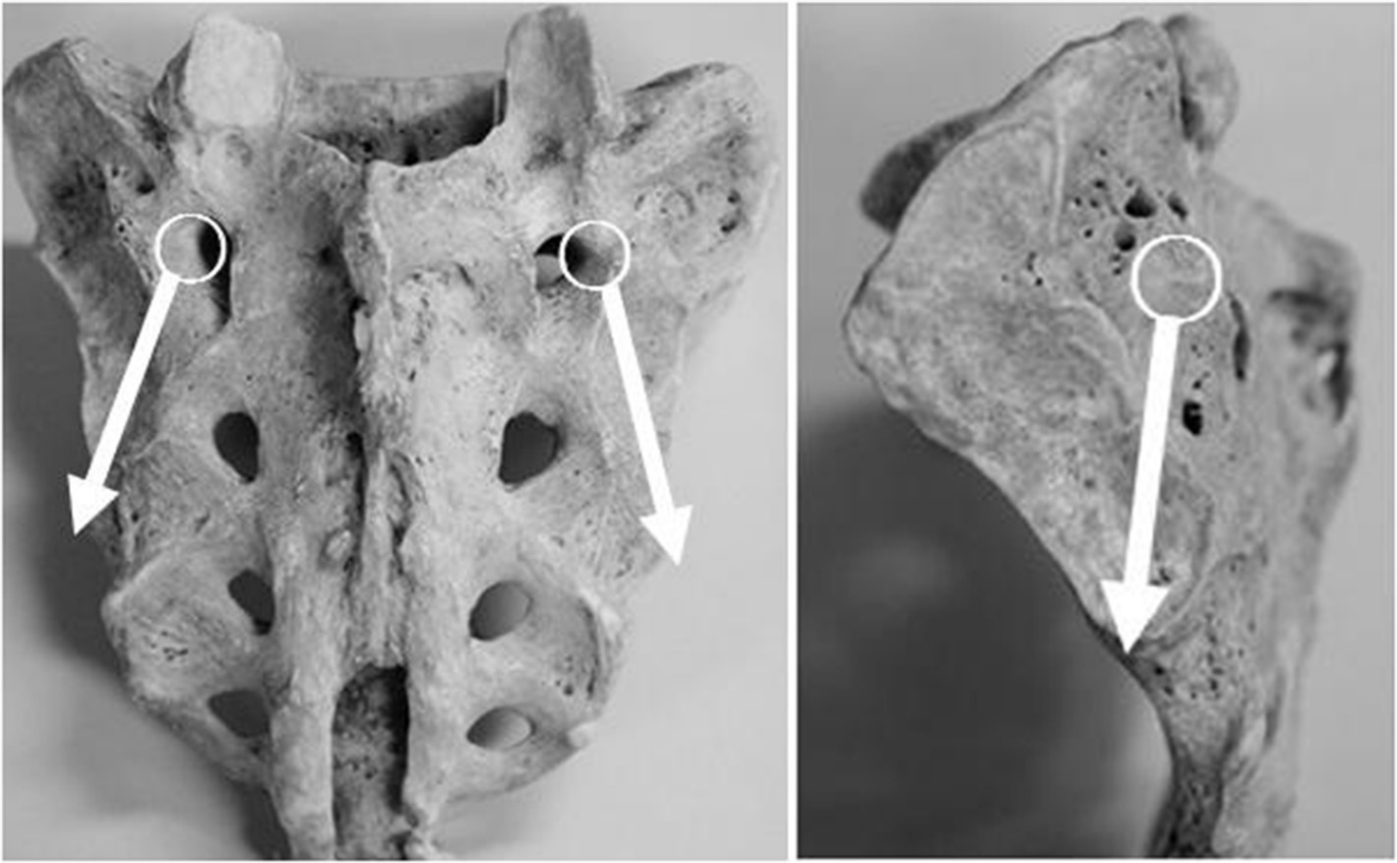
**White circles indicate entry points of the intrasacral screw.** Trajectories of the intrasacral screws are shown by arrows.

In this modified surgical technique, the patient is placed on the operating table in the prone position and a standard midline incision is placed over the spinal column down to the S2 level. The exposure is extended laterally to approximately 1 cm lateral to the first dorsal sacral foramen. Neurovascular structures exiting the foramen are cauterized due to exposure. The entry point of the intrasacral screw is made 1 mm lateral to the first dorsal sacral foramen. A burr or awl is used to make an entry hole at the cortex and a probe with a ball-shaped tip is gently inserted by hand pressure parallel to the sacroiliac joint into the cancellous bone toward the distal end of the joint. The aiming point of the distal end is located under the posterior inferior spine of the ilium. Fluoroscopy can be used to determine the distal end (Figure 1).

**Figure 2:**
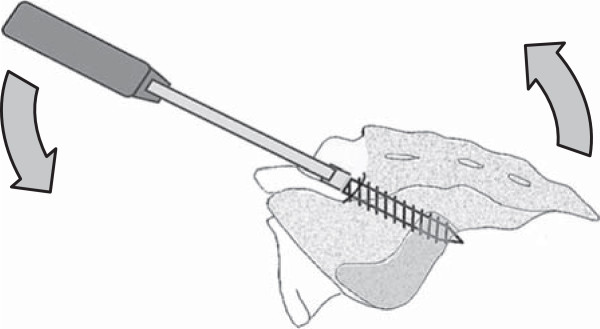
**Illustration of the intrasacral screw insertion.** Iliac buttress effect and stability of the intrasacral screw is confirmed by the flexural bending force through a screw inserter.

The surgeon must take care so that the probe is placed just under the subchondral bone of the sacroiliac joint. When the probe reaches the distal end of the lateral mass, the length of the inserted part of this probe is measured, and the length of the intrasacral screw is determined by adding 5 mm to the measured length of the inserted probe. A small perforation at the end of the lateral mass is made by light tapping of the probe with a hammer to set the bicortical screw purchase. Thereafter, a screw of an appropriate length is inserted and the stability of the screw in the sacrum is confirmed by flexural bending force using a screw inserter (Figure 2). Next, the S1 screw is placed after insertion of the intrasacral screw because the head of the S1 screw can block insertion of the intrasacral screw. The S1 screw is inserted to the promontory using the “tricortical” technique introduced by Lehman et al. [8]. Posterior lumbar interbody fusion is performed at segments L5/S and cephalad, if necessary. The rod is bent into an ideal curvature to correct the deformity and for easy assembly. The rod is then connected from the intrasacral and S1 screws to those cephalad using a cantilever maneuver for curve correction. Decortication of the laminae and bone grafting are performed to achieve additional posterior fusion. The muscle tissue is then released to cover the instrumentation and the wound is closed. The patients are instructed to restrict the range of hip motion for a few months, postoperatively.

### Instrumentation

We use intrasacral screws with polyaxial heads that allow a wide range of angulation formed between the head and screw axes; the ZODIAC® Spinal Fixation System (Alphatec Spine, Inc., Carlsbad, CA, USA) and the EXPEDIUM™ Dual Innie System (DePuy Synthes Spine, Inc., Raynham, MA, USA) or the CD HORIZON® SOLERA™ Spinal system (Medtronic, Inc. TN, USA). Screws are less than 7 mm in diameter because there is insufficient space in the lateral sacral mass in approximately 8% of patients to accommodate a 7-mm rod [7].

## Results

### Representative case presentation

#### Case 1

**Figure 3:**
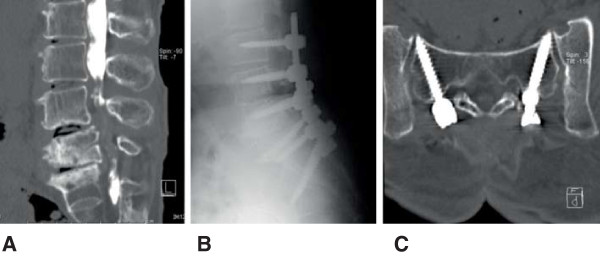
**Radiographs of a 67-year-old female with degenerative kyphosis and spinal canal stenosis in the lumbar spine. A**: Sagittal reconstruction image from the data of a computed tomography after the myelogram. **B**: Lateral view of the postoperative lumbar spine with instrumentation. **C**: Reconstructed image of the plane along the axes of intrasacral screws.

A 67-year-old female was consulted for a complaint of intermittent claudication within 15 min caused by low back pain and numbness in her bilateral legs. The patient’s history included spinal canal stenosis with spondylosis and kyphosis in the lumbar spine. Posterior spinal fusion was performed from L3 to the sacrum and interbody fusion was applied at levels L4/5 and L5/S. The surgical duration was 5 h and 28 min with an estimated blood loss of 929 mL. Autologous blood transfusion was used; however, allogeneic blood transfusion was not required. Lumbar lordosis was corrected from -7° to 31°. Walking was allowed with the use of a lumbosacral orthosis beginning on postoperative day 12. Lumbosacral fusion was achieved 1 year after the surgery, although proximal junctional kyphosis progressed 2 years after the surgery (Figure 3).

#### Case 2

**Figure 4:**
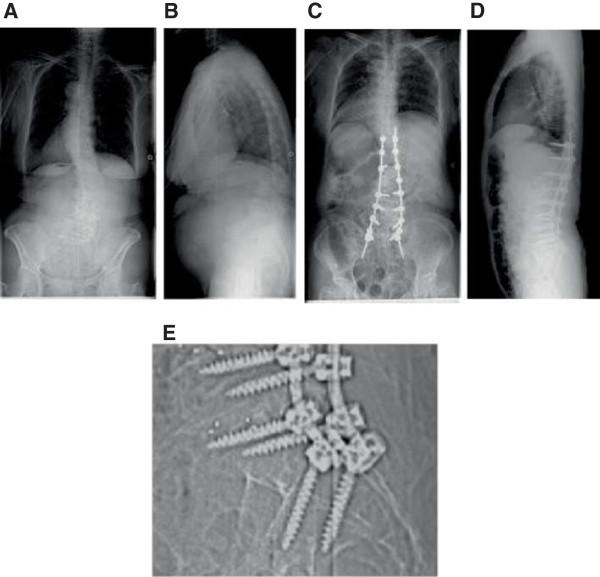
**Radiographs of a 77-year-old female with degenerative kyphoscoliosis. A**: Posteroanterior view of the preoperative whole spine. **B**: Lateral view of the preoperative whole spine. **C**: Posteroanterior view of the postoperative whole spine. **D**: Lateral view of the postoperative whole spine. **E**: Intrasacral screws are seen in the magnified view of the lumbosacral junction.

A 77-year-old female was admitted with a complaint of gait disturbance caused by low back pain and left-sided sciatica. She was unable to walk more than 20 m without the use of a cane. Her symptoms began to progress 10 years before. A preoperative X-ray exam revealed kyphoscoliosis of her lumbar spine with Cobb angles measuring 35° on the coronal view and -6° of lumbar lordosis. Posterior spinal fusion was performed from T11 to the sacrum using intrasacral screws. Interbody fusion was applied at disc levels L2/3, L3/4, L4/5, and L5/S, resulting in the correction of kyphoscoliosis with Cobb angles measuring 11°on a coronal view and 46°of lumbar lordosis. The duration of surgery was 6 h and 18 min with an estimated blood loss of 2362 mL. The patient received an autologous blood transfusion; however, allogenic transfusion was not required. There were no major intraoperative complications. Standing rehabilitation using a tilt table was started on postoperative day 4. Walking was allowed with the use of a thoracolumbosacral orthosis beginning on postoperative day 20. Her symptoms of low back pain and sciatica were markedly reduced, and she was able to walk for more than 10 min with the use of a cane 6 months after the surgery (Figure 4).

#### Case 3

**Figure 5:**
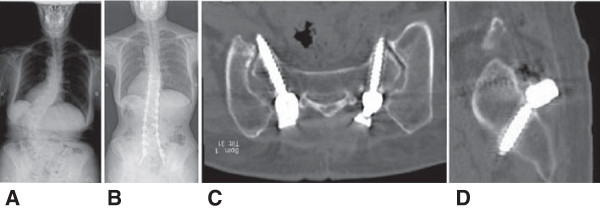
**Radiographs of a 72-year-old female with degenerative scoliosis. A**: Posteroanterior view of the preoperative whole spine. **B**: Posteroanterior view of the postoperative whole spine. **C**: Reconstructed image of the plane along the axes of the intrasacral screws. **D**: Reconstructed sagittal image of the plane along the axis of the intrasacral screw.

A 72-year-old female presented with a >20-year history of low back pain and radicular pain in her right leg. She was unable to walk without the assistance of a walker. Her preoperative Cobb angle was 67°on a coronal view. Posterior spinal fusion was performed from T9 to the sacrum using intrasacral screws. Interbody fusion was performed at disc levels L3/4, L4/5, and L5/S. Scoliosis was corrected to a Cobb angle of 18°. The duration of surgery was 7 h and 51 min with an estimated blood loss of 1271 mL. She received an autologous blood transfusion; however, allogenic transfusion was not required. There were no major intraoperative complications. Standing rehabilitation using a tilt table was started on postoperative day 5. Walking was allowed with the use of a thoracolumbosacral orthosis beginning on postoperative day 20. Her symptoms of low back pain and radicular pain were reduced, and she could walk for more than 10 min with the use of a cane 1 year after the surgery. Spinal fusion was confirmed, but slight loosening of the intrasacral screws was observed by computed tomography at follow-up (Figure 5).

## Discussion

The connection of an intrasacral rod to pedicle screws is one of the techniques for lumbosacral fixation reported by Jackson and McManus [7]. Theoretically, the ilium and sacroiliac ligaments provide a buttress effect to the rods against a flexural load. One of the difficulties of this procedure is in contouring the rod to fit the lateral sacral mass and spinal curvature. In addition, the procedure to assemble the rod to S1 and pedicle screws cephalad to it is technically demanding. Here we introduced an intrasacral screw as an alternative for a part of the rod. The use of this alternative technique with screws with polyaxial heads enables easier assembly of the rod and screws compared with previous techniques. This intrasacral screw technique is indicated for patients with kyphoscoliosis at the lumbosacral level.

Modification of rod insertion to the intrasacral screw has another advantage. The construct with an intrasacral screw is more stable than that with an intrasacral rod against pull- and/or back-out forces because the screw threads interdigitate with the subchondral bone under iliac buttress coverage with the dorsal and ventral cortex of the sacrum. Kuklo et al. [4] reported a similar modification of the Galveston iliac rod to the screw and Schwend et al. [9] performed a mechanical test in a cadaver model and demonstrated that iliac screws were more than three times stronger than iliac rods.

Posterior spinal fusion at the lumbosacral junction remains challenging because of the complex anatomy and poor bone quality of the sacrum [10]. Several techniques have been reported to achieve distal foundation of the instrumentation [11,12]. Sacroiliac screws [13] from the S2 alar crossing the sacroiliac joint to the ilium are considered to form a stronger foundation than the presented technique. However, the impact of sacroiliac screws on the sacroiliac joint remains uncertain in long-term follow-ups.

Limitations of this study include the small number of cases, short follow-up periods, and lack of biomechanical data. Furthermore, intrasacral screws may not provide a sufficient foundation for some patients who require a more extensive force to correct curvature. The combination of distal foundation techniques of instrumentation, use of an orthosis, and temporary limited postoperative activities can help to achieve successful fusion.

## Conclusion

The placement of an intrasacral rod by the Jackson and McManus technique can be modified to accommodate an intrasacral screw with a polyaxial head connected to the rod. This modification may contribute to stronger distal foundation and easy assembly of the instrumentation for posterior spinal fusion at the lumbosacral junction.

## Competing interests

Authors of this manuscript declare that they have no competing interests.

## Authors’ contribution

KF conceived the surgical modification and performed all the operations. MT was major contributor in writing the manuscript. MM coordinated the preparation of the manuscript. TA assisted with supervision and drafting of the manuscript. All authors read and approved the final manuscript.
